# Assessment of an enhanced program for depression management in primary care: a cluster randomized controlled trial. The INDI project (Interventions for Depression Improvement)

**DOI:** 10.1186/1471-2458-7-253

**Published:** 2007-09-20

**Authors:** Enric Aragonès, Antonia Caballero, Josep Ll Piñol, Germán López-Cortacans, Waleska Badia, Josep M Hernández, Pilar Casaus, Sílvia Folch, Josep Basora, Antonio Labad, the INDI research group

**Affiliations:** 1 Tarragona-Reus Primary Care Area, Catalan Health Institute, Spain; 2 University Psychiatric Hospital "Institut Pere Mata", Reus, Spain; 3 Unit of Psychiatry, Rovira i Virgili University, Reus, Spain; 4 Centre d'Atenció Primària de Constantí; Carrer dels Horts, 6. 43120 Constantí (Tarragona), Spain

## Abstract

**Background:**

Most depressed patients are attended at primary care. However, there are significant shortcomings in the diagnosis, management and outcomes of these patients. The aim of this study is to determine whether the implementation of a structured programme for managing depression will provide better health outcomes than usual management.

**Methods/Design:**

*Design*: A cluster-randomized controlled trial involving two groups, one of which is the control group consisting of patients who are treated for depression in the usual way and the other is the intervention group consisting of patients on a structured programme for treating depression.

*Setting*: 20 primary care centres in the province of Tarragona (Spain)

*Sample*: 400 patients over 18 years of age who have experienced an episode of major depression (DSM-IV) and who need to initiate antidepressant treatment

*Intervention*: A multi-component programme with clinical, educational and organisational procedures that includes training for the health care provider and evidence-based clinical guidelines. It also includes primary care nurses working as care-managers who provide educational and emotional support for the patients and who are responsible for active and systematic clinical monitoring. The programme aims to improve the primary care/specialized level interface.

*Measurements*: The patients will be monitored by telephone interviews. The interviewer will not know which group the patient belongs to (blind trial). These interviews will be given at 0, 3, 6 and 12 months.

*Main variables*: Severity of the depressive symptoms, response rate and remission rate.

*Analysis*: Outcomes will be analyzed on an intent-to-treat basis and the unit of analysis will be the individual patient. This analysis will take into account the effect of study design on potential lack of independence between observations within the same cluster.

**Discussion:**

The effectiveness of caring for depression in primary care can be improved by various strategies. The most effective models involve organisational changes and a greater role of nurses. However, these models are almost exclusively from the USA, and this randomized clinical trial will determine if this approach could be effective to improve the outcomes of depression in primary care in the Spanish health care system.

**Trial registration:**

ISRCTN16384353

## Background

Depressive disorders have been estimated to be the leading cause of disability (i.e. non-fatal burden) in the world [1]. Primary health care, which is the health system's first level of care, is the main ambit in which the most common mental disorders in the population are treatedf, including depression, and the majority of patients suffering from depression are attended exclusively at this care level [2]. However, it has been demonstrated that the detection, diagnosis, treatment and monitoring of patients with depression have significant shortcomings with regard to the model that could be referred to as "the best practice" [3].

In a recent study we found that 14.3% of consecutive primary care patients can be diagnosed with major depression according to DSM-IV criteria [4]. More than half of the depressed patients presented only somatic symptoms and were more difficult to detect by their primary care doctors than patients who openly revealed the psychological nature of their depression [5]. Furthermore, the fact that depression was detected was no guarantee that the patients would receive the appropriate treatment: although 72% of people suffering from depression were detected by their primary care doctor, only 34% received specific treatment with antidepressants while 48% were treated with anxiolytic or hypnotic drugs [6]. In primary care, the rates of non-compliance or early discontinuation of antidepressant prescriptions are high [7] and the evolution of the depressed patient once the treatment has been established is often inadequately monitored, if at all. Therefore, the opportunity to monitor therapeutic compliance and clinical evolution, and to implement measures to improve adherence to the treatment or to adjust inefficient treatments is lost. What is more, access to psychotherapeutic treatment of proven effectiveness as a first line therapy in certain types of mild or moderate depression within the ambit of primary care in the Spanish health system is almost non-existent. Each of these factors compromises the health outcomes that could be obtained in a depressed patient, according to scientific evidence.

In its mental health strategy, the World Health Organisation rightly considers that in order to reduce the impact of depression, in public health terms, the distance that exists between the availability of potentially effective therapeutic measures and the high proportion of depressed patients who are unable to benefit from such options must be overcome. A specific aim in this regard is to provide effective coverage for depression in primary care [8].

In order to improve the diagnosis and treatment of depression in primary care and obtain high rates of efficiency that are closer to the potential effectiveness of available treatments [9], certain strategies of an educational and organisational nature have been proposed, including the training of healthcare professionals, the availability of clinical practice guidelines and strategies to implement them, case management-allocating a central role to non-medical professionals, often nurses-, and collaboration between the primary care level and specialised psychiatric level. The complex models that involve organisational changes and a greater role for nurses rather than the implementation of simple measures (i. e. medical education or access to clinical guidelines as the only measures) have been shown to be most effective [10-12]. The principal limitation of the existing research is that the studies are almost exclusively from the United States, and this raises the question of whether strategies that are effective within one social context and health organisation can be equally effective in other countries with different healthcare structures.

## Methods/Design

### Objectives

The general aim is to determine whether a programme for treating depression leads to better health outcomes than the usual depression management provided in primary care in the Spanish healthcare system. The specific aims are to determine whether the programme for treating depression, compared to usual practice, will reduce the severity of depression, and increase the response and remission rates, and the degree of health-related quality of life at 3, 6 and 12 months.

### Design

**Figure 1:**
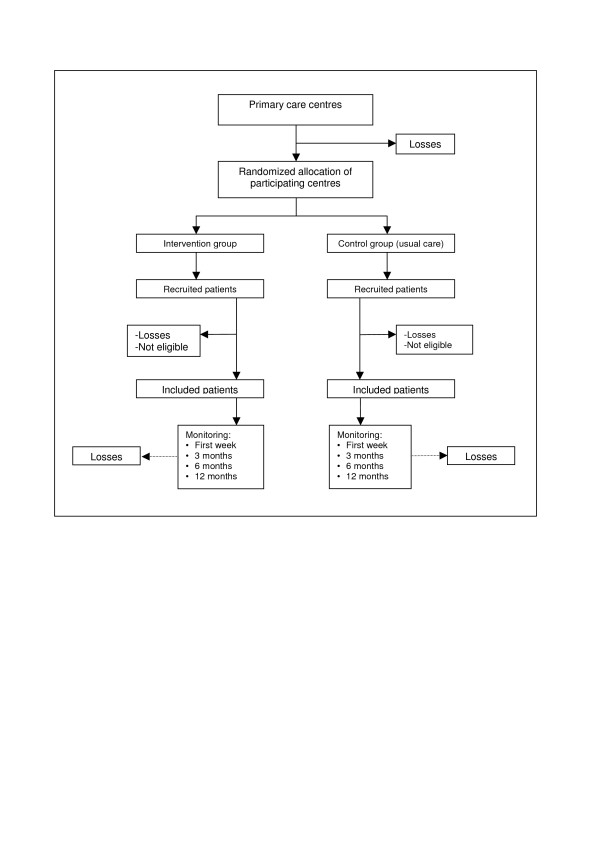
Flowchart: randomisation of centres, and sampling and monitoring of patients.

This is a controlled trial with a random allocation of clusters (primary healthcare centres) in two alternative branches (see Figure 1):

1. Usual management of depression (control group) and

2. Implementation of a programme to manage depression (intervention group).

The evaluation of the treatment outcomes will be done at patient level, evaluated individually [13].

### Setting and study sample

All of the 20 primary healthcare centres in the Tarragona-Reus Primary Care Area (Catalan Health Institute) in the province of Tarragona, Catalonia, Spain, have agreed to participate. The doctors from each centre who participated in the study had to be full-time employees, remain in the same location for the duration of the study and have a quota of patients assigned.

The doctors participating in the study will ask all patients who have suffered from an episode of major depression (DSM-IV) and who have been advised to take a new course of antidepressants to take part, until they reach their assigned quota of patients.

Patients considered for **inclusion **are those assigned to the doctor, aged ≥18 years, able to be contacted by telephone, who have been diagnosed with an episode of major depression (DSM-IV), have a score of >14 on the PHQ-9 (moderate-severe depression) or a score of 10 to 14 (mild depression) that has persisted for more than one month, and who have not received antidepressant medication in the previous three months.

The following patients will be **excluded**: those who suffer from physical, psychiatric or linguistic limitations or a concurrent illness that impede comprehension/participation in the study evaluations, patients with psychotic or bipolar disorders, patients with alcohol or drug dependence and patients who are pregnant or breastfeeding.

### Allocation of study groups

In order to ensure comparability between the intervention group and the control group, each centre will be paired with another centre with similar features [13]: urban/rural location, the number of participating doctors and the availability of a psychiatrist in the own centre. One of each pair of centres will be randomly allocated as a treatment or a control centre. The pairing and the allocation will be carried out by an independent person not involved in the study.

The centres agree to participate before the random allocation. The patients agree to participate without knowing to which study group their centre has been allocated.

### Intervention

The intervention consists of a multi-component programme that deals with depression based on published scientific evidence. It includes measures that can be reasonably applied within the Spanish health organisation. Such actions are of a clinical, training-based, organisational and health-related educational nature.

The programme includes tools for the systematic and structured evaluation and treatment of depression in primary care. There is an initial 8-hour course based on the clinical guidelines for treating depression recommended by the National Institute for Health and Clinical Excellence (NICE) [14] and designed to improve doctors' knowledge and skills in diagnosing depression, evaluating suicidal risk, clinical treatment, monitoring depression, and modifying the therapy in order to achieve remission in accordance with a treatment algorithm. The training puts the emphasis on the care procedure, active clinical monitoring of depressed patients and the options available when the proposed aims are not achieved (short-term remission and no relapse in the long term). The educational plan includes periodic updates.

A Depression Management Toolkit that contains a treatment algorithm for optimizing the prescription of antidepressant medication is available for the participating doctors.

The programme creates the role of case managers. The case managers are nurses on the staff of the participating primary care centres with specific training (a 8-hour initial course plus periodic updates) in the clinical aspects of depression, antidepressant treatment, secondary effects, treatment adherence and the methods to ensure this, warning signs in the evolution of depression, etc. The case-manager identifies individual, family, and community factors relevant to planning individualized care for depressed patients and their families, provides health education and support on health care needs and resources that help patients to recover or maintain their health and independence. The programme establishes the minimum number of nurse's visits with the patient: in the acute stage, initially one week after inclusion and then monthly until remission; in the continuation and maintenance stage the contacts will be every two/three months. However, the plan of follow-up visits is individualized depending on the nature of the patient and the evolution of the depression. The visits are structured and the patient is provided with information and education on the illness and the treatment, including "self-help" activities and health advice for the patients and their family members. Patients are provided with specifically designed printed and videographic material. Adherence to the therapeutic plan is systematically evaluated, the difficulties are identified so that compliance can be ensured, the possible adverse effects of the treatment are identified and the clinical evolution of the patient is evaluated through systematic use of the Patient Health Questionnaire Depression module (PHQ-9) [15]. All the information, including the score on the PHQ-9, is recorded and sent to the responsible doctors so that they have all the details on the condition and evolution of the patient and can use them to take decisions on the treatment (i. e. the need for changes to the treatment, treating side effects, re-evaluation, consultation with the psychiatrist, etc.).

To improve the primary care/specialised care interface, primary care doctors and psychiatrists are able to consult with each other by telephone or e-mail. Clear criteria for referrals to the specialised level will be established in order to improve the quality of referrals. In any case, whenever patient care is shared between primary and specialised professionals, responsibility for the treatment and monitoring of the patient will be clearly established to prevent any gaps in the care provided. The psychiatrists are given specific directives emphasising the care process and the therapeutic options for depressions that are resistant to treatment for the purpose of achieving short-term remission and preventing long-term relapse.

### Control group (usual management)

The doctors in the centres that continue with standard treatment use their own criteria to attend depressed patients and are allowed to use any resources they consider appropriate, including referral to the specialised level. Although the activities involving the detection and diagnosis of the depression are not included in the evaluation, the doctors in the intervention group could become more aware through the diagnosis of the depression and detect cases with a milder depression, introducing an element of bias in the inclusion of patients. In order to prevent this, the doctors in the control group are given a training session on diagnosing and detecting depression, with the same content as that of the doctors in the intervention group.

### Measurements

The results will be monitored and most of the data collected by means of standard questionnaires conducted by telephone interview by an independent qualified interviewer (a psychologist), who has been trained in telephone interview techniques and in psychiatric nosology. The interviewer will be unaware of which study group the patients being interviewed belong to ("blind"). The follow-up interviews shall take place at 0 (baseline), 3, 6 and 12 months after the inclusion of the patient.

#### Variables and instruments of measurement (See Table 1)

**Table 1:** Study variables

**Instrument**	**Assessment area**	**Applied by**	**Time(s) of assessments**
Sampling form	Age, sex, inclusion/exclusion criteria	Primary care physicians	Baseline
Sociodemographic data form	Age, sex, marital status, educational level, labour status, social class	Research interviewer by means of telephonic interview	Baseline
Duke Severity of Illness Checklist (DUSOI)	Global severity of physical comorbidity	Primary care physicians	Baseline
PRIME-MD; dysthymia and anxiety modules	Common psychiatric comorbidity	Primary care physicians	Baseline
Patient Health Questionnaire (PHQ-9)	Severity of depressive symptoms, remission and response rates	Research interviewer by means of telephone interview	Baseline, 3, 6 and 12 months
Questionnaire	Length of evolution of current depressive episode and previous history of depression.	Research interviewer by means of telephone interview	Baseline
SF-12 Health Survey	Health-related quality of life: mental health and physical health scores	Research interviewer by means of telephone interview	Baseline, 3, 6 and 12 months
Morisky-Green Test	Treatment adherence	Research interviewer by means of telephone interview	Baseline, 3, 6 and 12 months
Use of health resources questionnaire	Number of primary care, psychiatric and emergency visits, and hospitalisations owing to mental health problems	Research interviewer by means of telephone interview	3, 6 and 12 months
Satisfaction with the care received	Satisfaction of the patient with the clinical care got for his depression	Research interviewer by means of telephone interview	3, 6 and 12 months
Exploration of the computer database of pharmaceutical prescription and invoicing	Treatment with antidepressants. Medical prescription and consumption by the patient.	Research assistant	3, 6 and 12 months

##### Main outcome variables

In accordance with the aims of this study, the major outcome variables are response and remission rates and the measurement of depression severity as a continuous variable.

The severity of depressive symptoms will be measured by means of the PHQ-9 [15]. This scale is a brief self-reported diagnosis and a measure of the severity of major depression (DSM-IV). Several studies support its validity, feasibility, and its capacity to detect changes in depressive symptoms over time. A validated Spanish version is available [16]. Published data shows that telephone administration of the PHQ-9 is a reliable procedure for assessing depression [17].

Clinical remission should be the goal of acute treatment for depression [18]. It is defined as virtually complete relief of symptoms and return to full functioning, and is thought of as the optimal goal for the initial phase of treatment of depression [19]. We have adopted a PHQ-9 score of less than 5 as an operational indicator of remission [20].

Response is a defined as a 50% reduction in the severity of the symptoms measured with the PHQ-9 at baseline [15,19].

To measure health-related quality of life, we will use the SF-12 Health Questionnaire [21,22] which will provide two scores: one for physical health and one for mental health.

##### Secondary variables and effect modifiers

At baseline:

- The following sociodemographic data will be collected: sex, age, marital status (single, married/coupled, divorced/separated or widowed), education (no studies, primary, lower secondary, upper secondary and university), labour status, and social class (I, II, IIIN, IIIM, IV and V of the British Registrar General's Scale) [23].

- The severity of the physical comorbidity will be measured using the Duke Severity of Illness Checklist (DUSOI) [24,25]. This checklist will be filled in by each patient's family physician. For each diagnosis of a physical nature, a score is assigned to the symptoms, complications, prognosis and expected response to treatment. The overall severity of the patient, evaluated from 0 to 100, is obtained from an equation that gives a greater coefficient to the main diagnosis and successively lower coefficients to the other diagnoses.

- To assess the most common psychiatric comorbidity in depressed patients we will use the dysthymia and anxiety sections of the Primary Care Evaluation of Mental Disorders (PRIME-MD). This is a 2-stage rapid screening and interview procedure that can generate a range of diagnoses of mental disorders according to DSM-IV criteria [26,27].

- We will establish how long the current depressive episode has been evolving and the previous history of depression.

In the follow-up interviews, besides measuring depressive symptomatology with the PHQ-9 and health-related quality of life with the SF-12 Health Questionnaire:

- Therapeutic compliance will be evaluated by the Morisky-Green Test [28], the self-report compliance (Haynes-Sackett test) [29], and searching the computer databases for pharmaceutical prescription and invoicing.

- The use of health resources in the evaluated period is determined by means of interviewing the patient: number and type of primary care, psychiatric and emergency visits and hospitalisations for mental health problems.

- The patients' satisfaction with the care received is evaluated using a single item (a Likert scale with five response options) [30].

### Statistical methods

#### Sample size

To calculate the sample size, we consider the remission rate at six months as the main result variable.

On the basis of published research data [31], we assume that this will be 30% in the control group and we aim to detect a difference of 16% or more between this group and the intervention group.

Accepting an alpha risk of 0.05 and a beta risk of <0.20 in a bilateral contrast and assuming a 15% loss in continuing the treatment, we would need 169 subjects in each group in a simple random sampling.

To correct this figure for design effect (cluster randomisation) [13,32] we shall use the formula:

Deff = 1 + (m - 1) × ICC

where Deff: design effect, m: size of clusters and ICC: intraclass coefficient correlation.

Provisionally assuming an ICC = 0.01 and m = 20, the Deff will be 1.19. Thus, each group needs to consist of 201 subjects (1.19 × 169 = 201) divided into 10 clusters of 20 patients.

We have no reliable data on the ICC in the sample and in the variables we studied and we have provisionally used a prudent figure that will be checked once the data is available.

#### Analysis strategy

Randomisation is performed at primary care centre level and the results of the treatment will be analysed at individual patient level [13]. The analyses shall be per intent to treat.

First we will compare the intervention group with the control group in order to verify that there are no significant differences between the two groups (socio-demographic data, clinical baseline data, etc.). We shall use the mean (Standard Deviation) in the continuous variables and percentages in the categorical variables. For comparisons we shall use the Student-T test for continuous variables and the Chi-squared test for categorical variables. Non-parametric tests may also be used.

The main variables of the result are the depressive symptomatology (PHQ-9 score), response to treatment (reduction of 50% or more in the baseline score), remission (score of <5) and quality of life related to health (SF-12 score) at 3, 6 and 12 months.

Process variables include the number of visits for depression to the primary care centre, psychiatrist, emergencies and hospitalisations and the continued use of anti-depressants over 3 months.

We shall use the analysis of the linear mixed models of the SPSS v.15 statistical package, including the two effects, fixed and random, to analyse the effect of the continuous result variables (depression symptomatology [PHQ-9 score], quality of life related to health [SF-12 score]). We shall analyse the effect of the treatment on the categorical result variables (response and remission rates) by analysing the general linear models of the SPSS v.15. The random effects of these linear models (mixed and general) provide the structure that enables us to take into account the effect of randomisation by clusters owing to a potential lack of independence between observations within the same centre [33].

## Ethical aspects

The design of the study, with the random allocation of primary care centres to the control group or intervention group, makes it necessary to obtain informed consent on two levels: firstly, that of the participating doctors before they are allocated to the intervention or the control groups and, secondly, that of the participating patients, obviously once their centre has been allocated to the corresponding group, but before they are aware which group it is.

Before they give their consent, the patients are provided with a general overview of the aims and activities of the study. They are also informed that they will be participating voluntarily, and that they can choose to drop out at any time with the guarantee that they will continue to receive the treatment considered most appropriate by their doctor. The patients in the control group will receive the treatment considered most suitable by their doctor, without limitations.

The information provided to the health professionals is similar: participation in the study is voluntary and they can choose to drop out without any negative repercussions for the health or healthcare of their patients if they consider that continued participation will cause them harm. The participating professionals will sign a document in which they state that they will provide their patients with information, that they have no conflict of interest and that participation in the study favours the interests of their patients.

If the treatment evaluated proves to be effective, patients in all participating centres, including those in the control group, are guaranteed continued access to it. In this respect, the competent management authority will give their guarantee before the study begins and the centres are informed of it when their participation is requested.

The Study Protocol was approved by the Research Ethics Committee of the Jordi Gol i Gurina Primary Care Research Institute (IDIAP), Barcelona, on March 29, 2006 (ref: P06/16).

## Forecast execution dates

Initial recruitment of patients: June 2007

Deadline for recruitment of patients: December 2008

Deadline for period of patient monitoring: December 2009

Publication of the results: March 2010

## Discussion

Previous experiences, particularly in the United States, have shown that the implementation of multifactorial programmes for the care of depression can give rise to better health results [10-12] and at present in several European health systems there are several research projects in progress that aim to investigate the usefulness of similar models for handling depression [34-36]. Along these lines, the objective of the INDI project is to create a programme for handling depression that can be applied in primary health care in the Spanish health system and to evaluate its effectiveness.

The intervention programme consists of an integral package of a variety of measures (organization for systematic and structured handling of depression, case management, professional training, clinical guidelines and treatment algorithm, psychological education of the patients) and one of its qualities is that it has been designed bearing in mind that it should be applicable to real caring practice so that, if the evaluation results are favourable, it can be easily generalized. The programme does not require considerable resources and the main aim of organizational measures is to optimize existing ones.

One of the innovative aspects, in our ambit, is to define and promote the role of the nurse in the systematic and structured handling of depression. The nurse is given the role of case manager and a central role in organizing the care of depressed patients. In an attempt to make the most of the resources available, it was decided to use the nurses already on the staff of the primary health care centre instead of contracting external case managers.

One of the limitations of our model is that it focuses on pharmacological treatment and does not consider psychotherapy as a front-line therapeutic option in primary care because in "real" caring practice in the Spanish health system it is a resource that is not often available and, although it would have been possible to include it in the context of a research project, it would be difficult to generalize in practice.

In the design of the clinical trial we opted to randomize clusters because the intervention to be evaluated is designed for primary health care centres (e.g. organizational measures) and health professionals (e.g. medical education) while the results of these interventions will be measured in individual patients in the form of health outcomes. This design makes it possible to avoid the attenuation of the effect of the intervention due to the possible contamination between study groups if the randomization were carried out individually and the patients assigned to the intervention and control groups were attended in the same place and even by the same professionals.

## Abbreviations

Deff, Design effect; DSM-IV, Diagnostic and Statistical Manual of Mental Disorders, Fourth Edition; DUSOI, Duke Severity of Illness Checklist; ICC, Intraclass coefficient correlation; IDIAP, Primary Care Research Institute (in Catalan: Institut D'Investigació en Atenció Primària); NICE, National Institute for Health and Clinical Excellence; PHQ-9, Patient Health Questionnaire; PRIME-MD, Primary Care Evaluation of Mental Disorders; SF-12, Medical Outcomes Study Health Survey, Short-Form, 12 items

## Competing interests

The authors declare that they have no competing interests.

## Authors' contributions

EA is the principal investigator and developed the original idea for the study. The study design was further developed by EA, AC, JLP and GL. The following have intervened in the design and the planning of the intervention that is evaluated: EA, AC, SF, JB and JMH (training of the participating doctors, support materials – Depression Management Toolkit); WB and GL (nurses' interventions), JMH and EA (patient health education), PC and AL (primary care/specialised level interface). JLP developed the statistical methods. All authors have read and corrected draft versions, and approved the final version.

## Pre-publication history

The pre-publication history for this paper can be accessed here:


